# Supplementary material for: Evaluation of a scheme to identify risks for tail biting in pigs
Source: PLoS One. 2024 Aug 29;19(8):e0305960. doi: 10.1371/journal.pone.0305960 (PMC11361435; doi:10.1371/journal.pone.0305960)
Supplement: S1 File — (PDF) [file pone.0305960.s001.pdf]

ANIMAL HEALTH IRELAND

Contributing to a profitable and sustainable farming and agri-food sector through improved animal health

Assessment and Management of Risk Factors in Tail-biting in Pig Production

Farmer Name:

28072020 PIGS TaiBitRis FORM v1.1

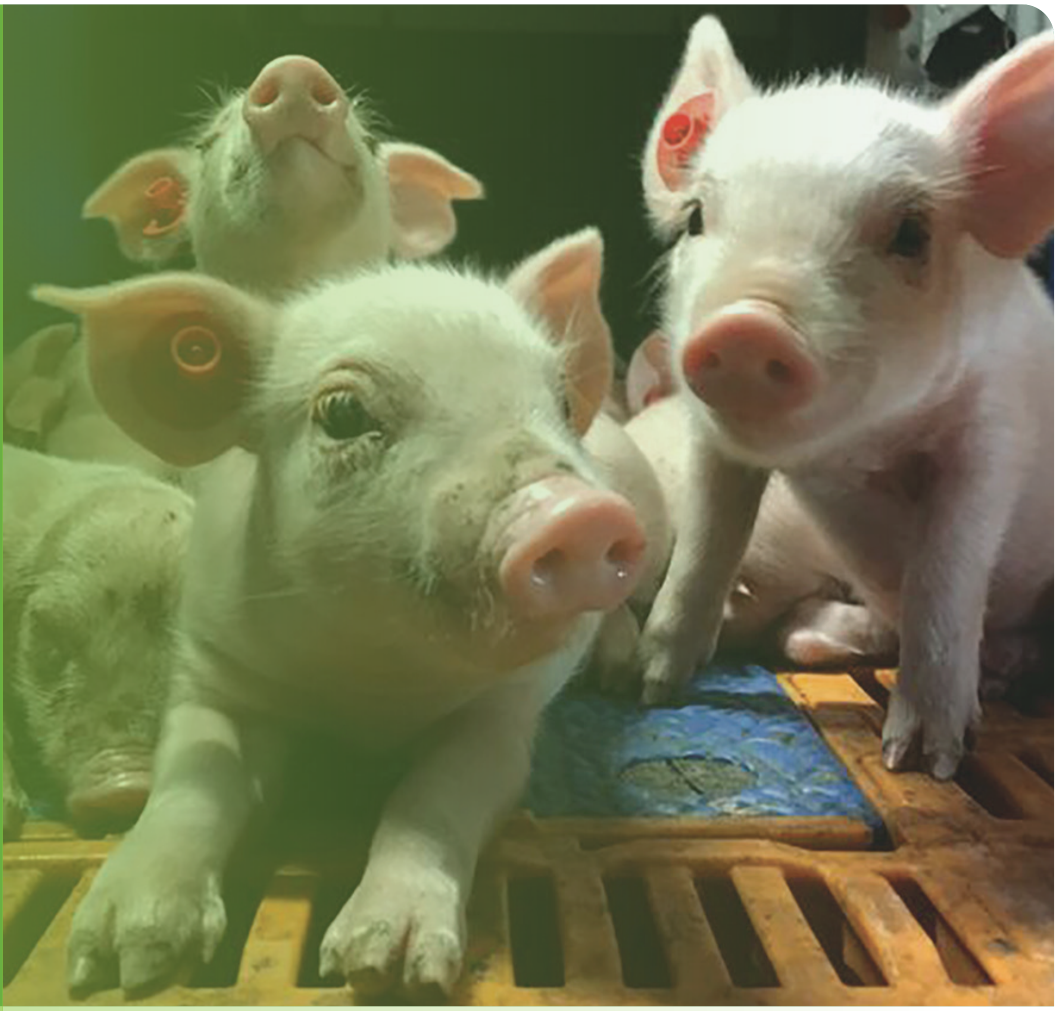

www.animalhealthireland.ie

NATIONAL PIG HEALTH PROGRAMME

Animal Health Ireland, 4-5 The Archways, Carrick-on-Shannon, Co. Leitrim, N41 WN27

Pig HealthCheck
AnimalHealthIreland.ie

Ireland's European Structural and Investment Funds Programmes 2014-2020
Co-funded by the Irish Government and the European Union

The European Agricultural Fund for Rural Development
Europe investing in rural areas

SECTION A: 1 of 6

SECTION	
Weaner House	
Fattening House	
Dry Sow House	
Post Service	
House Farrowing House	
Gilt House	
Boar House	
Hospital House	
Other	

PIG TYPE			
Weaner Stage 1		Farrowing sows	
Weaner Stage 2		Boars	
Fatteners		Gilts	
Breeding Sows		Piglets	
Dry sows			

PEN ID#									
PEN LOCATION									
NUMBER OF PIGS IN THE PEN									
PEN LENGTH (M)				M					
PEN WIDTH (M)				M					
SLATTED FLOOR	0%		1-25%		26-50%		51-75%		76-100%
SEX	Male		Female		Mixed				
FINAL WEIGHT ACHIEVED BY PIGS IN THIS PEN				KG (Avg)					
TAIL LENGTH	All docked		All long		Mixed				
CAN ALL PIGS FEED AT THE SAME TIME?	Yes		No						
HOW MANY DRINKERS ARE IN THE PEN?									

HAVE THE PIGS IN THIS PEN AN EFFECTIVE VACCINATION PROGRAMME?	
There is an effective vaccination programme for animals in this house	
An improved vaccination programme needs to be considered for animals in this house	
Vaccination for animals in this house is not sufficient to address ongoing health deficits	

			Examples (as per Commission working document)	Other examples
COUNT THE NUMBER OF TYPES OF ITEMS IN THE PEN FOR EACH CATEGORY		Optimal	Straw, green fodder (hay, grass, silage, alfalfa, etc.), miscanthus pressed or chopped, root vegetables when used as bedding	
		Sub-optimal	Peanut shells, ground wood, ground maize corn cobs, natural ropes, compressed straw cylinders, pellets, hessian cloth, shredded paper or natural soft rubber as bedding, or optimal (above) in a rack	Sawdust, woodchips, fresh soft wood planks (e.g. pine, spruce), branches of fresh wood
		Marginal	e.g. objects, such as hard plastic piping or chains.	Toys (hanging or on floor), tyres, empty cans/oil drums, balls (hanging at end of chain or on floor), hard wood planks or pieces of wood at end of chain, synthetic ropes
		None		

COUNT THE NUMBER OF PIGS IN THE PEN WITH:								
	Tucked tails				Flank lesions (circular)			
	Injured tails				Aggression lesions (straight)			
	Injured or imperfect ears				Dirty flanks/haunches			

RECORD OF PIG BEHAVIOURS

Stand quietly and for a 5 minute period record the number of occasions that each of the following behaviours defined below is observed in the pen

**Damaging behaviour**	This is oral (mouth/nose) behaviour that is directed towards another pigs' body, which can cause physical damage or pain, but is not motivated by aggression. The behaviour can range from gentle slow chewing to focused bites (usually to the ears or tails). Damaging behaviour can also take the form of sustained nosing of a body part (usually the flanks) resulting in a circular lesion. We have sub-divided it into tail, ear, flank and 'other' directed behaviours.
**Fixtures and fittings**	This is oral behaviour that is directed towards any physical part of the pen such as walls, floors, feeders, drinkers or pen dividers that are not designed to be rooted at or chewed by the pigs
**Enrichment**	This refers to any interaction the pigs direct towards the enrichment provided, including chewing, sniffing, nosing, rooting, pushing etc.
**Aggressive biting**	This behaviour is motivated by aggression, and unlike damaging behaviour, is normally associated with swift, forward movements by the 'attacking' pig accompanied by quick snapping movements by the jaws (i.e. open mouthed). The attacking pig may even chase the victim pig. The behaviour is normally directed towards the front part of another pig (although it can be directed towards the rear if the attacker is aiming to get access to a resource such as the feeder).

**BEHAVIOUR CATEGORY**<br>Damaging behaviour	**Count**	**Total**
Category (Tail, Ear etc.)	卌 I	6
Tail		
Ear		
Other		
Fixtures and fittings		
Enrichment		
Aggressive biting		

**BASED ON YOUR OBSERVATIONS OF BEHAVIOURS AND BODY LESIONS OBSERVED IN THIS PEN IS THERE A RISK OF TAIL BITING FOR THESE PIGS?**	Yes	
	No	

**RISK VALUES**	
**Risk Statement**	**Risk Value**
Not Observed	**0**
Risk Category Statement is correct	**1**
I was not able to identify risks associated with this Risk Category	**2**
I have identified that risk exists for this Risk Category	**3**
There are clearly risks associated with this Risk Category	**4**

VETERINARY OPINION ON MEASURES ASSOCIATED WITH TAIL BITING RISK FOR THIS PEN

**Risk Category**	**RISK VALUE 0-4**
Environmental Enrichment provision represents no risk for tail biting	
There is adequate thermal comfort and air quality for these pigs	
The health of these pigs provides no risk of tail biting	
Competition issues for the pigs in this pen do not give rise to risks for tail biting	
The pen design and use for these pigs does not present risk for tail biting	
Feeding processes for these pigs do not contribute to risks for tail biting for these pigs	

MANAGEMENT RECOMMENDATIONS

To reduce factors considered to increase the risk of tail-biting and other aggressive behaviours at this stage of production.

1.

2.

3.

Other Comments:

SECTION A: 2 of 6

SECTION	
Weaner House	
Fattening House	
Dry Sow House	
Post Service	
House Farrowing House	
Gilt House	
Boar House	
Hospital House	
Other	

PIG TYPE			
Weaner Stage 1		Farrowing sows	
Weaner Stage 2		Boars	
Fatteners		Gilts	
Breeding Sows		Piglets	
Dry sows			

PEN ID#										
PEN LOCATION										
NUMBER OF PIGS IN THE PEN										
PEN LENGTH (M)				M						
PEN WIDTH (M)				M						
SLATTED FLOOR	0%		1-25%		26-50%		51-75%		76-100%	
SEX	Male		Female		Mixed					
FINAL WEIGHT ACHIEVED BY PIGS IN THIS PEN				KG (Avg)						
TAIL LENGTH	All docked		All long		Mixed					
CAN ALL PIGS FEED AT THE SAME TIME?	Yes		No							
HOW MANY DRINKERS ARE IN THE PEN?										

HAVE THE PIGS IN THIS PEN AN EFFECTIVE VACCINATION PROGRAMME?	
There is an effective vaccination programme for animals in this house	
An improved vaccination programme needs to be considered for animals in this house	
Vaccination for animals in this house is not sufficient to address ongoing health deficits	

			Examples (as per Commission working document)	Other examples
COUNT THE NUMBER OF TYPES OF ITEMS IN THE PEN FOR EACH CATEGORY		Optimal	Straw, green fodder (hay, grass, silage, alfalfa, etc.), miscanthus pressed or chopped, root vegetables when used as bedding	
		Sub-optimal	Peanut shells, ground wood, ground maize corn cobs, natural ropes, compressed straw cylinders, pellets, hessian cloth, shredded paper or natural soft rubber as bedding, or optimal (above) in a rack	Sawdust, woodchips, fresh soft wood planks (e.g. pine, spruce), branches of fresh wood
		Marginal	e.g. objects, such as hard plastic piping or chains.	Toys (hanging or on floor), tyres, empty cans/oil drums, balls (hanging at end of chain or on floor), hard wood planks or pieces of wood at end of chain, synthetic ropes
		None		

COUNT THE NUMBER OF PIGS IN THE PEN WITH:							
Tucked tails				Flank lesions (circular)			
Injured tails				Aggression lesions (straight)			
Injured or imperfect ears				Dirty flanks/haunches			

RECORD OF PIG BEHAVIOURS

Stand quietly and for a 5 minute period record the number of occasions that each of the following behaviours defined below is observed in the pen

**Damaging behaviour**	This is oral (mouth/nose) behaviour that is directed towards another pigs' body, which can cause physical damage or pain, but is not motivated by aggression. The behaviour can range from gentle slow chewing to focused bites (usually to the ears or tails). Damaging behaviour can also take the form of sustained nosing of a body part (usually the flanks) resulting in a circular lesion. We have sub-divided it into tail, ear, flank and 'other' directed behaviours.
**Fixtures and fittings**	This is oral behaviour that is directed towards any physical part of the pen such as walls, floors, feeders, drinkers or pen dividers that are not designed to be rooted at or chewed by the pigs
**Enrichment**	This refers to any interaction the pigs direct towards the enrichment provided, including chewing, sniffing, nosing, rooting, pushing etc.
**Aggressive biting**	This behaviour is motivated by aggression, and unlike damaging behaviour, is normally associated with swift, forward movements by the 'attacking' pig accompanied by quick snapping movements by the jaws (i.e. open mouthed). The attacking pig may even chase the victim pig. The behaviour is normally directed towards the front part of another pig (although it can be directed towards the rear if the attacker is aiming to get access to a resource such as the feeder).

**BEHAVIOUR CATEGORY** Damaging behaviour	Count	Total
Category (Tail, Ear etc.)	~~IIII~~ I	6
Tail		
Ear		
Other		
Fixtures and fittings		
Enrichment		
Aggressive biting		

**BASED ON YOUR OBSERVATIONS OF BEHAVIOURS AND BODY LESIONS OBSERVED IN THIS PEN IS THERE A RISK OF TAIL BITING FOR THESE PIGS?**	Yes	
	No	

**RISK VALUES**	
**Risk Statement**	**Risk Value**
Not Observed	0
Risk Category Statement is correct	1
I was not able to identify risks associated with this Risk Category	2
I have identified that risk exists for this Risk Category	3
There are clearly risks associated with this Risk Category	4

VETERINARY OPINION ON MEASURES ASSOCIATED WITH TAIL BITING RISK FOR THIS PEN

**Risk Category**	**RISK VALUE 0-4**
Environmental Enrichment provision represents no risk for tail biting	
There is adequate thermal comfort and air quality for these pigs	
The health of these pigs provides no risk of tail biting	
Competition issues for the pigs in this pen do not give rise to risks for tail biting	
The pen design and use for these pigs does not present risk for tail biting	
Feeding processes for these pigs do not contribute to risks for tail biting for these pigs	

MANAGEMENT RECOMMENDATIONS
To reduce factors considered to increase the risk of tail-biting and other aggressive behaviours at this stage of production.

1.

2.

3.

Other Comments:

SECTION A: 3 of 6

SECTION	
Weaner House	
Fattening House	
Dry Sow House	
Post Service	
House Farrowing House	
Gilt House	
Boar House	
Hospital House	
Other	

PIG TYPE			
Weaner Stage 1		Farrowing sows	
Weaner Stage 2		Boars	
Fatteners		Gilts	
Breeding Sows		Piglets	
Dry sows			

PEN ID#	
PEN LOCATION	
NUMBER OF PIGS IN THE PEN	
PEN LENGTH (M)	M
PEN WIDTH (M)	M
SLATTED FLOOR	0% ☐ 1-25% ☐ 26-50% ☐ 51-75% ☐ 76-100% ☐
SEX	Male ☐ Female ☐ Mixed ☐
FINAL WEIGHT ACHIEVED BY PIGS IN THIS PEN	KG (Avg)
TAIL LENGTH	All docked ☐ All long ☐ Mixed ☐
CAN ALL PIGS FEED AT THE SAME TIME?	Yes ☐ No ☐
HOW MANY DRINKERS ARE IN THE PEN?	

HAVE THE PIGS IN THIS PEN AN EFFECTIVE VACCINATION PROGRAMME?	
There is an effective vaccination programme for animals in this house	
An improved vaccination programme needs to be considered for animals in this house	
Vaccination for animals in this house is not sufficient to address ongoing health deficits	

COUNT THE NUMBER OF TYPES OF ITEMS IN THE PEN FOR EACH CATEGORY		Examples (as per Commission working document)	Other examples
	Optimal	Straw, green fodder (hay, grass, silage, alfalfa, etc.), miscanthus pressed or chopped, root vegetables when used as bedding	
	Sub-optimal	Peanut shells, ground wood, ground maize corn cobs, natural ropes, compressed straw cylinders, pellets, hessian cloth, shredded paper or natural soft rubber as bedding, or optimal (above) in a rack	Sawdust, woodchips, fresh soft wood planks (e.g. pine, spruce), branches of fresh wood
	Marginal	e.g. objects, such as hard plastic piping or chains.	Toys (hanging or on floor), tyres, empty cans/oil drums, balls (hanging at end of chain or on floor), hard wood planks or pieces of wood at end of chain, synthetic ropes
	None		

COUNT THE NUMBER OF PIGS IN THE PEN WITH:			
Tucked tails		Flank lesions (circular)	
Injured tails		Aggression lesions (straight)	
Injured or imperfect ears		Dirty flanks/haunches	

RECORD OF PIG BEHAVIOURS

Stand quietly and for a 5 minute period record the number of occasions that each of the following behaviours defined below is observed in the pen

**Damaging behaviour**	This is oral (mouth/nose) behaviour that is directed towards another pigs' body, which can cause physical damage or pain, but is not motivated by aggression. The behaviour can range from gentle slow chewing to focused bites (usually to the ears or tails). Damaging behaviour can also take the form of sustained nosing of a body part (usually the flanks) resulting in a circular lesion. We have sub-divided it into tail, ear, flank and 'other' directed behaviours.
**Fixtures and fittings**	This is oral behaviour that is directed towards any physical part of the pen such as walls, floors, feeders, drinkers or pen dividers that are not designed to be rooted at or chewed by the pigs
**Enrichment**	This refers to any interaction the pigs direct towards the enrichment provided, including chewing, sniffing, nosing, rooting, pushing etc.
**Aggressive biting**	This behaviour is motivated by aggression, and unlike damaging behaviour, is normally associated with swift, forward movements by the 'at-tacking' pig accompanied by quick snapping movements by the jaws (i.e. open mouthed). The attacking pig may even chase the victim pig. The behaviour is normally directed towards the front part of another pig (although it can be directed towards the rear if the attacker is aiming to get access to a resource such as the feeder).

**BEHAVIOUR CATEGORY** Damaging behaviour	**Count**	**Total**
Category (Tail, Ear etc.)	~~IIII~~ I	6
Tail		
Ear		
Other		
Fixtures and fittings		
Enrichment		
Aggressive biting		

**BASED ON YOUR OBSERVATIONS OF BEHAVIOURS AND BODY LESIONS OBSERVED IN THIS PEN IS THERE A RISK OF TAIL BITING FOR THESE PIGS?**		
	Yes	
	No	

**RISK VALUES**	
**Risk Statement**	**Risk Value**
Not Observed	0
Risk Category Statement is correct	1
I was not able to identify risks associated with this Risk Category	2
I have identified that risk exists for this Risk Category	3
There are clearly risks associated with this Risk Category	4

VETERINARY OPINION ON MEASURES ASSOCIATED WITH TAIL BITING RISK FOR THIS PEN

**Risk Category**	**RISK VALUE 0-4**
Environmental Enrichment provision represents no risk for tail biting	
There is adequate thermal comfort and air quality for these pigs	
The health of these pigs provides no risk of tail biting	
Competition issues for the pigs in this pen do not give rise to risks for tail biting	
The pen design and use for these pigs does not present risk for tail biting	
Feeding processes for these pigs do not contribute to risks for tail biting for these pigs	

MANAGEMENT RECOMMENDATIONS

To reduce factors considered to increase the risk of tail-biting and other aggressive behaviours at this stage of production.

1.

2.

3.

Other Comments:

SECTION A: 4 of 6

SECTION	
Weaner House	
Fattening House	
Dry Sow House	
Post Service	
House Farrowing House	
Gilt House	
Boar House	
Hospital House	
Other	

PIG TYPE			
Weaner Stage 1		Farrowing sows	
Weaner Stage 2		Boars	
Fatteners		Gilts	
Breeding Sows		Piglets	
Dry sows			

PEN ID#									
PEN LOCATION									
NUMBER OF PIGS IN THE PEN									
PEN LENGTH (M)				M					
PEN WIDTH (M)				M					
SLATTED FLOOR	0%		1-25%		26-50%		51-75%		76-100%	
SEX	Male		Female		Mixed				
FINAL WEIGHT ACHIEVED BY PIGS IN THIS PEN				KG (Avg)					
TAIL LENGTH	All docked		All long		Mixed				
CAN ALL PIGS FEED AT THE SAME TIME?	Yes		No						
HOW MANY DRINKERS ARE IN THE PEN?									

HAVE THE PIGS IN THIS PEN AN EFFECTIVE VACCINATION PROGRAMME?	
There is an effective vaccination programme for animals in this house	
An improved vaccination programme needs to be considered for animals in this house	
Vaccination for animals in this house is not sufficient to address ongoing health deficits	

		Examples (as per Commission working document)	Other examples
COUNT THE NUMBER OF TYPES OF ITEMS IN THE PEN FOR EACH CATEGORY	Optimal	Straw, green fodder (hay, grass, silage, alfalfa, etc.), miscanthus pressed or chopped, root vegetables when used as bedding	
	Sub-optimal	Peanut shells, ground wood, ground maize corn cobs, natural ropes, compressed straw cylinders, pellets, hessian cloth, shredded paper or natural soft rubber as bedding, or optimal (above) in a rack	Sawdust, woodchips, fresh soft wood planks (e.g. pine, spruce), branches of fresh wood
	Marginal	e.g. objects, such as hard plastic piping or chains.	Toys (hanging or on floor), tyres, empty cans/oil drums, balls (hanging at end of chain or on floor), hard wood planks or pieces of wood at end of chain, synthetic ropes
	None		

COUNT THE NUMBER OF PIGS IN THE PEN WITH:								
	Tucked tails				Flank lesions (circular)			
	Injured tails				Aggression lesions (straight)			
	Injured or imperfect ears				Dirty flanks/haunches			

RECORD OF PIG BEHAVIOURS

Stand quietly and for a 5 minute period record the number of occasions that each of the following behaviours defined below is observed in the pen

**Damaging behaviour**	This is oral (mouth/nose) behaviour that is directed towards another pigs' body, which can cause physical damage or pain, but is not motivated by aggression. The behaviour can range from gentle slow chewing to focused bites (usually to the ears or tails). Damaging behaviour can also take the form of sustained nosing of a body part (usually the flanks) resulting in a circular lesion. We have sub-divided it into tail, ear, flank and 'other' directed behaviours.
**Fixtures and fittings**	This is oral behaviour that is directed towards any physical part of the pen such as walls, floors, feeders, drinkers or pen dividers that are not designed to be rooted at or chewed by the pigs
**Enrichment**	This refers to any interaction the pigs direct towards the enrichment provided, including chewing, sniffing, nosing, rooting, pushing etc.
**Aggressive biting**	This behaviour is motivated by aggression, and unlike damaging behaviour, is normally associated with swift, forward movements by the 'attacking' pig accompanied by quick snapping movements by the jaws (i.e. open mouthed). The attacking pig may even chase the victim pig. The behaviour is normally directed towards the front part of another pig (although it can be directed towards the rear if the attacker is aiming to get access to a resource such as the feeder).

**BEHAVIOUR CATEGORY** Damaging behaviour	Count	Total
Category (Tail, Ear etc.)	~~IIII~~ I	6
Tail		
Ear		
Other		
Fixtures and fittings		
Enrichment		
Aggressive biting		

**BASED ON YOUR OBSERVATIONS OF BEHAVIOURS AND BODY LESIONS OBSERVED IN THIS PEN IS THERE A RISK OF TAIL BITING FOR THESE PIGS?**	Yes	
	No	

**RISK VALUES**	
**Risk Statement**	**Risk Value**
Not Observed	0
Risk Category Statement is correct	1
I was not able to identify risks associated with this Risk Category	2
I have identified that risk exists for this Risk Category	3
There are clearly risks associated with this Risk Category	4

VETERINARY OPINION ON MEASURES ASSOCIATED WITH TAIL BITING RISK FOR THIS PEN

**Risk Category**	**RISK VALUE 0-4**
Environmental Enrichment provision represents no risk for tail biting	
There is adequate thermal comfort and air quality for these pigs	
The health of these pigs provides no risk of tail biting	
Competition issues for the pigs in this pen do not give rise to risks for tail biting	
The pen design and use for these pigs does not present risk for tail biting	
Feeding processes for these pigs do not contribute to risks for tail biting for these pigs	

MANAGEMENT RECOMMENDATIONS

To reduce factors considered to increase the risk of tail-biting and other aggressive behaviours at this stage of production.

1.

2.

3.

Other Comments:

SECTION A: 5 of 6

SECTION	
Weaner House	
Fattening House	
Dry Sow House	
Post Service	
House Farrowing House	
Gilt House	
Boar House	
Hospital House	
Other	

PEN ID#			

PEN LOCATION	

NUMBER OF PIGS IN THE PEN			

PEN LENGTH (M)				M

PEN WIDTH (M)				M

SLATTED FLOOR	0%		1-25%		26-50%		51-75%		76-100%	

SEX	Male		Female		Mixed	

FINAL WEIGHT ACHIEVED BY PIGS IN THIS PEN				KG (Avg)

TAIL LENGTH	All docked		All long		Mixed	

CAN ALL PIGS FEED AT THE SAME TIME?	Yes		No	

HOW MANY DRINKERS ARE IN THE PEN?		

PIG TYPE			
Weaner Stage 1		Farrowing sows	
Weaner Stage 2		Boars	
Fatteners		Gilts	
Breeding Sows		Piglets	
Dry sows			

HAVE THE PIGS IN THIS PEN AN EFFECTIVE VACCINATION PROGRAMME?	
There is an effective vaccination programme for animals in this house	
An improved vaccination programme needs to be considered for animals in this house	
Vaccination for animals in this house is not sufficient to address ongoing health deficits	

			Examples (as per Commission working document)	Other examples
COUNT THE NUMBER OF TYPES OF ITEMS IN THE PEN FOR EACH CATEGORY		Optimal	Straw, green fodder (hay, grass, silage, alfalfa, etc.), miscanthus pressed or chopped, root vegetables when used as bedding	
		Sub-optimal	Peanut shells, ground wood, ground maize corn cobs, natural ropes, compressed straw cylinders, pellets, hessian cloth, shredded paper or natural soft rubber as bedding, or optimal (above) in a rack	Sawdust, woodchips, fresh soft wood planks (e.g. pine, spruce), branches of fresh wood
		Marginal	e.g. objects, such as hard plastic piping or chains.	Toys (hanging or on floor), tyres, empty cans/oil drums, balls (hanging at end of chain or on floor), hard wood planks or pieces of wood at end of chain, synthetic ropes
		None		

COUNT THE NUMBER OF PIGS IN THE PEN WITH:							
	Tucked tails				Flank lesions (circular)		
	Injured tails				Aggression lesions (straight)		
	Injured or imperfect ears				Dirty flanks/haunches		

RECORD OF PIG BEHAVIOURS
Stand quietly and for a 5 minute period record the number of occasions that each of the following behaviours defined below is observed in the pen

**Damaging behaviour**	This is oral (mouth/nose) behaviour that is directed towards another pigs' body, which can cause physical damage or pain, but is not motivated by aggression. The behaviour can range from gentle slow chewing to focused bites (usually to the ears or tails). Damaging behaviour can also take the form of sustained nosing of a body part (usually the flanks) resulting in a circular lesion. We have sub-divided it into tail, ear, flank and 'other' directed behaviours.
**Fixtures and fittings**	This is oral behaviour that is directed towards any physical part of the pen such as walls, floors, feeders, drinkers or pen dividers that are not designed to be rooted at or chewed by the pigs
**Enrichment**	This refers to any interaction the pigs direct towards the enrichment provided, including chewing, sniffing, nosing, rooting, pushing etc.
**Aggressive biting**	This behaviour is motivated by aggression, and unlike damaging behaviour, is normally associated with swift, forward movements by the 'attacking' pig accompanied by quick snapping movements by the jaws (i.e. open mouthed). The attacking pig may even chase the victim pig. The behaviour is normally directed towards the front part of another pig (although it can be directed towards the rear if the attacker is aiming to get access to a resource such as the feeder).

▸ **BEHAVIOUR CATEGORY** Damaging behaviour	**Count**	**Total**
Category (Tail, Ear etc.)	~~IIII~~ I	6
Tail		
Ear		
Other		
Fixtures and fittings		
Enrichment		
Aggressive biting		

▸ **BASED ON YOUR OBSERVATIONS OF BEHAVIOURS AND BODY LESIONS OBSERVED IN THIS PEN IS THERE A RISK OF TAIL BITING FOR THESE PIGS?**		
	Yes	
	No	

**RISK VALUES**	
**Risk Statement**	**Risk Value**
Not Observed	**0**
Risk Category Statement is correct	**1**
I was not able to identify risks associated with this Risk Category	**2**
I have identified that risk exists for this Risk Category	**3**
There are clearly risks associated with this Risk Category	**4**

▸ **VETERINARY OPINION ON MEASURES ASSOCIATED WITH TAIL BITING RISK FOR THIS PEN**	
**Risk Category**	**RISK VALUE 0-4**
Environmental Enrichment provision represents no risk for tail biting	
There is adequate thermal comfort and air quality for these pigs	
The health of these pigs provides no risk of tail biting	
Competition issues for the pigs in this pen do not give rise to risks for tail biting	
The pen design and use for these pigs does not present risk for tail biting	
Feeding processes for these pigs do not contribute to risks for tail biting for these pigs	

▸ **MANAGEMENT RECOMMENDATIONS**
To reduce factors considered to increase the risk of tail-biting and other aggressive behaviours at this stage of production.

1.

2.

3.

Other Comments:

SECTION A: 6 of 6

SECTION	
Weaner House	
Fattening House	
Dry Sow House	
Post Service	
House Farrowing House	
Gilt House	
Boar House	
Hospital House	
Other	

Weaner Stage 1		Farrowing sows	
Weaner Stage 2		Boars	
Fatteners		Gilts	
Breeding Sows		Piglets	
Dry sows			

PEN ID#										
PEN LOCATION										
NUMBER OF PIGS IN THE PEN										
PEN LENGTH (M)				M						
PEN WIDTH (M)				M						
SLATTED FLOOR	0%		1-25%		26-50%		51-75%		76-100%	
SEX	Male		Female		Mixed					
FINAL WEIGHT ACHIEVED BY PIGS IN THIS PEN				KG (Avg)						
TAIL LENGTH	All docked		All long		Mixed					
CAN ALL PIGS FEED AT THE SAME TIME?	Yes		No							
HOW MANY DRINKERS ARE IN THE PEN?										

HAVE THE PIGS IN THIS PEN AN EFFECTIVE VACCINATION PROGRAMME?	
There is an effective vaccination programme for animals in this house	
An improved vaccination programme needs to be considered for animals in this house	
Vaccination for animals in this house is not sufficient to address ongoing health deficits	

			Examples (as per Commission working document)	Other examples
COUNT THE NUMBER OF TYPES OF ITEMS IN THE PEN FOR EACH CATEGORY		Optimal	Straw, green fodder (hay, grass, silage, alfalfa, etc.), miscanthus pressed or chopped, root vegetables when used as bedding	
		Sub-optimal	Peanut shells, ground wood, ground maize corn cobs, natural ropes, compressed straw cylinders, pellets, hessian cloth, shredded paper or natural soft rubber as bedding, or optimal (above) in a rack	Sawdust, woodchips, fresh soft wood planks (e.g. pine, spruce), branches of fresh wood
		Marginal	e.g. objects, such as hard plastic piping or chains.	Toys (hanging or on floor), tyres, empty cans/oil drums, balls (hanging at end of chain or on floor), hard wood planks or pieces of wood at end of chain, synthetic ropes
		None		

COUNT THE NUMBER OF PIGS IN THE PEN WITH:								
	Tucked tails				Flank lesions (circular)			
	Injured tails				Aggression lesions (straight)			
	Injured or imperfect ears				Dirty flanks/haunches			

RECORD OF PIG BEHAVIOURS

Stand quietly and for a 5 minute period record the number of occasions that each of the following behaviours defined below is observed in the pen

**Damaging behaviour**	This is oral (mouth/nose) behaviour that is directed towards another pigs' body, which can cause physical damage or pain, but is not motivated by aggression. The behaviour can range from gentle slow chewing to focused bites (usually to the ears or tails). Damaging behaviour can also take the form of sustained nosing of a body part (usually the flanks) resulting in a circular lesion. We have sub-divided it into tail, ear, flank and 'other' directed behaviours.
**Fixtures and fittings**	This is oral behaviour that is directed towards any physical part of the pen such as walls, floors, feeders, drinkers or pen dividers that are not designed to be rooted at or chewed by the pigs
**Enrichment**	This refers to any interaction the pigs direct towards the enrichment provided, including chewing, sniffing, nosing, rooting, pushing etc.
**Aggressive biting**	This behaviour is motivated by aggression, and unlike damaging behaviour, is normally associated with swift, forward movements by the 'at-tacking' pig accompanied by quick snapping movements by the jaws (i.e. open mouthed). The attacking pig may even chase the victim pig. The behaviour is normally directed towards the front part of another pig (although it can be directed towards the rear if the attacker is aiming to get access to a resource such as the feeder).

**BEHAVIOUR CATEGORY** Damaging behaviour	**Count**	**Total**
Category (Tail, Ear etc.)	~~||||~~ |	6
Tail		
Ear		
Other		
Fixtures and fittings		
Enrichment		
Aggressive biting		

**BASED ON YOUR OBSERVATIONS OF BEHAVIOURS AND BODY LESIONS OBSERVED IN THIS PEN IS THERE A RISK OF TAIL BITING FOR THESE PIGS?**		
	Yes	
	No	

**RISK VALUES**	
**Risk Statement**	**Risk Value**
Not Observed	0
Risk Category Statement is correct	1
I was not able to identify risks associated with this Risk Category	2
I have identified that risk exists for this Risk Category	3
There are clearly risks associated with this Risk Category	4

VETERINARY OPINION ON MEASURES ASSOCIATED WITH TAIL BITING RISK FOR THIS PEN

**Risk Category**	**RISK VALUE 0-4**
Environmental Enrichment provision represents no risk for tail biting	
There is adequate thermal comfort and air quality for these pigs	
The health of these pigs provides no risk of tail biting	
Competition issues for the pigs in this pen do not give rise to risks for tail biting	
The pen design and use for these pigs does not present risk for tail biting	
Feeding processes for these pigs do not contribute to risks for tail biting for these pigs	

MANAGEMENT RECOMMENDATIONS
To reduce factors considered to increase the risk of tail-biting and other aggressive behaviours at this stage of production.

1.

2.

3.

Other Comments:

SECTION B: TASAH (PIGS) FARM – REVIEW DECLARATION FORM

VISIT DATE	D	D	M	M	Y	Y	Y	Y

HERD OWNER	
Full Name	
Pig Herd Number	
Address	
Email Address	
Phone Number	

ENTERPRISE TYPE	Birth To Bacon	Fattening	Wean To Finish	Breeder to Weaner

VETERINARY PRACTITIONER		
Name		
Vet Reg Number (#/Year)		(#/YEAR)
Practice Name		
Practice Address		
Email Address		
Mobile Number		

DECLARATION - HERDOWNER/SENIOR RESPONSIBLE PERSON

By signing, I confirm as/on behalf of the herdowner, the above visit took place, that the information provided is accurate to the best of my knowledge and that I consent to the captured data being shared with Animal Health Ireland, Department of Agriculture, Food and the Marine, Teagasc and the Irish Cattle Breeding Federation (subcontracted by AHI to build and maintain the Pig HealthCheck database), for the purposes of managing the TASAH programme and my participation within it, providing farm management feedback, benchmarking and beyond the lifetime of the TASAH programme for scientific and research purposes. I am aware of my rights under the General Data Protection Regulations (available through the 'GDPR' link on AHI's homepage at www.animalhealthireland.ie).

**Herdowner's Signature**		Date	D	D	M	M	Y	Y	Y	Y

DECLARATION - VETERINARY PRACTITIONER

By signing, I confirm that: the above stated visit took place; all submitted data is accurate to the best of my knowledge; that where risks associated with tail biting in pigs in one or more pens were identified that I have agreed recommendations to address these with the farmer (as itemized per pen). I understand that I must, 1) Upload the results to the AHI CRM webform, 2) Provide a copy of the management recommendations captured in the document to the herdowner, 3) Provide a completed, legible copy of this declaration page to AHI (hard/soft copy), to be eligible for payment. I consent to the sharing of my Vet Reg No. and name (Animal Health Ireland, the Department of Agriculture Food and the Marine and Irish Cattle Breeding Federation) and contact details for the purposes of completing administration (inc. payment) around this review and management and evaluation of the Pig HealthCheck programme with Teagasc for the purposes of further research and am aware of my rights under the General Data Protection Regulations (available through the 'GDPR' link on AHI's website homepage at www.animalhealthireland.ie).

**Veterinary Practitioner's Signature**		Date	D	D	M	M	Y	Y	Y	Y
