# Supplementary material for: Evaluation of a scheme to identify risks for tail biting in pigs
Source: PLoS One. 2024 Aug 29;19(8):e0305960. doi: 10.1371/journal.pone.0305960 (PMC11361435; doi:10.1371/journal.pone.0305960)
Supplement: Attachment — Submitted filename: Response to Reviewers.docx [file pone.0305960.s002.docx]

Review Comments to the Author

Please use the space provided to explain your answers to the questions above. You may also include additional comments for the author, including concerns about dual publication, research ethics, or publication ethics. (Please upload your review as an attachment if it exceeds 20,000 characters)

Reviewer #1: The submitted manuscript describing the evaluation of a tool to identify risks for tail biting in pigs fails to provide data to support the conclusions. The statistical analysis is not well described nor appropriate such that the results cannot be interpreted with any confidence. Therefore, any conclusions are not supported. There appears to be many instances of missing data.

AU: Thank you for your feedback and specific comments, and we hope that the manuscript is now easier to follow and review.

Lines 282-288: the provided descriptive statistics are difficult to understand. Only the second paragraph refers to management practices, so move the heading (4.2 Management practices) and insert an additional heading 4.1 Descriptive statistics.

AU: We have changed the headings to 4.1 Farm description, and 4.2 Management practices (Line 334).

Example: does Line 283 indicate that the finisher stage sample population was 85 pens with 37.4±58.6 pigs/pen?

AU: Yes, the line did indicate that over the total of 85 pens assessed in the finisher stage the average of pigs per assessed pens were 37.4±58.6 pigs per pen. This text has now been redrafted for clarity (Line 335).

Example: what do the Line 287 numbers after 6421 and Line 288 numbers after 7197 refer to?

AU: We have clarified the text in this paragraph (now L340 to 341).

Lines 289-290: delete "A summary of the management practices observed over all pens can be seen in Figure 3."

AU: The sentence was deleted accordingly.

Line 290: insert "(Figure 3)" after "99%"

AU: The sentence was updated accordingly. Now line 344.

Line 295: replace "that were all" with "tails"

AU: The sentence was changed to clarify that all pigs were tail docked (line 348).

Lines 298-299: delete "A breakdown of the category type and amount of enrichment observed in the pens is provided in Table 4."

AU: The sentence was deleted accordingly.

Line 300: insert "(Table 4)" after "items"

AU: The sentence was changed accordingly. The table becomes Table 5 (Line 352).

Line 311: delete "Table 5 shows the level of risk assigned by the assessors with relation to each risk category"

AU: The sentence was deleted accordingly.

Line 312: replace "minimum" with "minor"

AU: The sentence was changed accordingly (line 370).

Line 314: insert "(Table 5)" after "system"

AU: The sentence was changed accordingly (line 372). Now Table 6

Lines 315-316: delete "Table 6 reported the level of risk assigned for those pens where assessors reported no risk for tail biting."

AU: The sentence was deleted accordingly.

Line 317: insert "(Table 6)" after "factors"

AU: The sentence was changed accordingly (line 372).

Table 5: Please explain the percentages. Based on the title, values between 0 and 2 would be expected.

AU: Now Table 6. We have changed the wording of the title and hope that it is now clearer. “Table 6. The proportion of times each level of risk was assigned to each of the risk categories by the assessors (n = 158 pens)”

Line 326: change heading to "4.4 Skin damage"

AU: The heading was changed to “4.4. Tail skin damage” (Line 389).

Lines 327-330: Is the data presented somewhere in the manuscript?

AU: The raw data are available as supplementary material. We have changed the wording of the paragraph as follow: “At slaughter, 69.6% of the pig tails presented no tail damage (score 0), 15.5% presented minor skin damage to the tail tip (score 1), 11% presented evidence of teeth marks (score 2), 3% presented moderate damage (score 3), and only 1% presented severe tail skin damage (score 4). At farm level, minor skin damage was always most prevalent, and ranged from ≥ 83% to 100% of carcases. Moderate damage ranged from ≥1% to ≤ 14% of carcases, and severe damage from ≤1% to ≤4% of carcases.” (lines 389-395)

Lines 334-337: Indicate the variation in the values that the statistics report. Reduce the 2 significant figures used in Line 335 to only 1 significant figures as in Line 337 for consistency.

Lines 338-339: If the data presented somewhere, please indicate. If not, please provide the values.

Line 350: Figure 5 only shows pig production stage, so remove "the amount of enrichment provision"

Line 353: more what?

AU: This section has been changed considerably in line with our revised statistics, and the results are now in graphical format (Figures 4 and 5). From line 396 to 422

Line 365: the data is not in Table 2

AU: Apologies for this typo, we meant Table 4. Now line 427.

Line 375: indicate the association value

AU: We have edited this paragraph so that the data presented reflects the management factors associated with the Risk Factor ‘Pen Layout’ (Lines 442-444).

Lines 377-379: are Feeding System and feeding space confounded?

AU: Now line 445. The two terms in fact refer to different things, and we hope that due to the significant re-writing we have carried out that this is now clear. ‘Feeding System’ refers to a ‘Risk category’, and ‘feeding space’ refers to the assessors’ evaluation as to whether or not the pigs can feed simultaneously.

Lines 384-385: delete "Table 7 shows the number of lesions found by the assessors for each body condition listed in the risk protocol, divided according to whether the risk of tail biting was detected."

AU: The sentence was deleted accordingly.

Line 388: insert "(Table 7)" after "not"

AU: This section has been re-worded considerably, and we hope that it is now more clear. From line 448 to line 456. Table 8 was then added to the section (line 458).

Line 387: how was "no difference" determined?

AU: This was determined by statistically comparing the proportion of animals that had physical impairments in each pen, in the pens that were deemed to have a risk of tail biting, and the pens that were deemed not to have a risk. This is described in the statistics section, L283-295. We have updated the results section to show the data and levels of significance.

Lines 397-398: Is the data presented somewhere in the manuscript?

AU: Apologies for the omission, these data are now included. We also noticed a slight error in the coding for the analysis, so some of the previously identified differences are no longer significant. Now lines 459-462

Lines 417-546: were not carefully reviewed due to limitations in the results discussed previously

AU: We hope that now that we have improved the materials and methods and statistics sections, that the reviewer will now feel confident in assessing the discussion. The discussion starts now at line 486

Figures 4-7. The standard error values seem very large and should be checked. All the values in the figures should be labeled to indicate statistical differences. Providing a single letter does not indicate which values are different from each other.

AU: We have updated the figures with the re-analysed data, and have improved their structure so that differences between means are more clearly indicated.

Reviewer #2: In their study, D’Alessio et al. Investigate the utility of a tail biting risk assessment tools for veterinarians. This tool includes a classification of several management and health xxx in combination with observations of behaviours to assess the risk of tail biting on farm. The risk assessment of trained veterinarians was compared to actual lesions recorded at the slaughterhouse and found to be not reliable. The authors provide a range of reasons why the tool failed in their article. Although the results provide a valuable overview of the prevalence of tail biting and reflect management practices in Ireland, they appear to be of limited interest to other countries with different assessment tools, particularly as the authors do not suggest any major improvements to the tool or training.

AU: Thank you for your comment. We have added new content to the discussion to address this issue. To support assessors and improve the training period, we suggested to prologue the training period, including the use online training material. An evaluation of the Private Veterinary Practitioners’ experience in implementing the tool would also support future adaptions of the tool, to improve its implementation (L600-602). We have also referred to a current study to adapt another more detailed Risk Assessment tool to assess hazards and protective factors on Irish pigs’ farms, and now we are in the process to validate it (L661-688).

In general, the tool is insufficiently described. Without prior extended knowledge of the WebHat protocol, the protocol is difficult to understand. The authors should be clearer in explaining how the risk categories are assigned from the parameters evaluated. For each level of the parameter, it should be made clear how this impacts the assessment of risk, for example if there is only marginal enrichment how does this translate in assessment of risk value? Does marginal enrichment mean a risk of 4 in the category? What are the expectations regarding, for instance, vaccination status or mixing of sexes, and tail biting?

AU: The link to the WebHat is provided in the introduction at L69. To try and improve clarity further however we have added text to the materials and methods to explain better the definitions of the levels of risk, and how they should be applied (L134 – 141). It transpired that the assessors used the levels in a different way than instructed, and we have explained that from L235-241. This is why we grouped the risk levels in the analysis. It is discussed further in the discussion, and likely contributed to why the tool proved ineffective in identifying the specific risk factors for the assessed farms included in the study. (L549-552 and L560-602)

Throughout the results it is not enough to indicate (P<0.05) we need more information on the model output with information on the type of model, estimate, degrees of freedom, etc. This can be in the results section but also in a table. Same for the correlation outputs, just indicating rho values is not enough.

AU: We have improved the information provided in the results section which now provides the information requested. (L333-480)

Material and methods

Line 119: The assigned values should be explained in the text and not only in the table. Please also give more details as to how the assessment was conducted for each category.

AU: The section was improved significantly, and we hope that the description of risk categories and levels of risk that could be applied are more clear (L134 to 152).

Line 169: It is unclear to the reader what the sample size refers to, please clarify whether this is the number of animals per year or farm where you got permission for examination.

AU: The sentence was modified to clarify that the sample size refers to the number of farms assessed in each year (L192-194).

Line 212: consider using “grouped” instead of “collapsed”.

AU: This paragraph was re-written to improve clarity as to which welfare indicators were used in analysis (Line 238 - 244).

Line 213: there is one “and” too much in the parenthesis, consider replacing it with a comma

AU: See reply to previous comment. We have placed inverted commas around ‘fixtures and fittings’ throughout the manuscript to clarify that this was one item on the original ethogram.

Line 220-221: Here, the associations between the risks and their indicators are presented; however in table 3 it is written “indicators hypothesised to be associated with each risk category” which is not specified in the text. I find this confusing as in the next line it says that only indicators associated with a risk factor were included in the analysis but it is unclear how these associations were decided and whether they are real or predicted.

AU: The decision as to which indicators and management practices were selected was based upon the experience of the authors, which in itself is based upon the literature, and their own research into tail biting (L260-262). KO has managed a research programme in this area for 10 years, AH has previously supervised PhD and MSc students in the area of risk factors associated with tail lesions, and CG has extensive experience of consulting with Private Veterinary Practitioners and industry personnel in the area of risk factors for tail biting.

Table 3: please provide information on how to interpret this table. It is not clear to me, for example, how dirty flanks are indicative of thermal comfort (or the lack of it).

AU: Now Table 4. Dirty flanks were selected as a welfare indicator as pigs are known to huddle when too cold, and wallow when too warm, which can increase the risk of rubbing dirt on each others bodies. We have added some additional explanation to the legend.

Line 240 and 265: Please specify if the model was linear or generalized linear; in the latter case, which distribution was used.

AU: We have clarified that linear models were used.

Line 242: I assume these indicators were the response variable, but I feel this could be made clearer. To me it seems there was a separate model for each of these variables with management practices as fixed factors and pen as a random effect, is this accurate?

AU: Yes, this is correct. We have re-phrased this paragraph and hope that you find it clearer (L283 – 295).

Line 245 and 246: factors instead of effects? I assume the number of pigs per drinker was also a fixed effect, but a continuous one.

AU: See above

Line 275: Tendency toward significance does not represent statistical significance and therefore should not be discussed as it can lead to over interpretation.

AU: We agree, and have deleted all reference to tendencies.

Line 277: The nature of the transformations needs to be specified for each variable they were necessary and for each model.

AU: No data were transformed, and as such we have deleted this sentence.

Table 4: it is unclear what the categories mean, provide more detail in the text. For instance, is it the number or the type of enrichment objects that are classified as “suboptimal” etc.?

AU: More clarification was added to the table in the form of footnotes.

Line 308: What level of risk value does “considered to be a risk” refer to? It would be good to specify clearly if this is from risk 1, or 2. As I mentioned above, the risks levels are not well explained and make this difficult to understand. “assessors” instead of “assessor’s”.

AU: This refers to what is described in L144-152, whereby assessors were asked to consider whether they think there is an overall level of risk of tail biting in the pen. Grammar has been corrected.

Line 312: Please define in the methods what is the level of minimum risk. The risk category levels as explained in the methods are not clear enough to establish what is the minimum reported.

AU: We have added more description to the materials and methods regarding the levels of risk. It was up to the professional opinion of the assessor, without specific guidance, if not the statements reported in the tool, to assign the risk level. We agree that the definitions are not clear. However these were inserted by an external party who was also involved in the development of the tool, without piloting the definitions with either potential assessors or other personnel involved in the tool development.

Table 5: Minor and Major risks need to be defined in the methods. From my example above I do not see how risk 1 is a minor risk from enrichment as risk 1 says the statement “Environmental enrichment provision represents no risk for tail biting” is correct. No risk for tail biting is not minor risk. Additionally, why are risk 3 and 4 not mentioned?

AU: The data was condensed in this fashion upon the advice of the third author of the manuscript, who has responsibility for liaising with assessors and co-ordinating training. Assessors were consulted, and they informed the authors that they considered both level one and 2 to imply that there was minimal risk for tail biting in the pen. Levels 3 and 4 were being applied interchangeably whenever a risk factor related to that risk category could be clearly identified (e.g. a low enrichment allowance, is a clear risk for ‘Enrichment’; single space feeders are a clear risk factor related to the category ‘Feeding processes’). This is why we have defined them as minor and major for the rest of the analysis.

Table 6: does this table include the first row of Table 5? It is unclear what the table shows, and, in particular, what the difference is to Table 5.

AU: This is now Table 7. We have improved the legend to make clear that the data in this table refers only to the sub sample of pens where there was no overall level of risk assigned to the pen.

Figures 4-6: Difficult to interpret the letter, what are the comparisons? Why “P<0.05” in Fig. 7? There is no letter indicating statistically significant difference?

AU: We have updated and improved the quality of the figures, so we hope that they are easier to understand

Line 368: provide p-value with coefficient

AU: The P-Value was added as required (P<0.05). Now line 428

Discussion

The discussion is well written and I mostly agree with it. However, it is often mentioned that some observations and findings are contrary to the literature, because conditions prevented the assessors from measuring the variable correctly, or because insufficient training resulted in a lack of reporting of certain variables (such as injuries). In this case, however, this makes the evaluation rather unreliable, because if the assessors could not assess the different variables correctly, then it is difficult to draw any conclusions from this study.

It seems to me that the method was not standardized enough and allowed for too much interpretation by the assessors, which led to results that were ultimately uninformative.

Unfortunately, the discussion is confined to describing the situation and explaining the results, but do not suggest how to proceed or how to solve the problem, which would have made the manuscript of broader interest.

AU: Thank you for your comment. We added a new section on the conclusion paragraph (Lines 651-666) where we explained that due to the poor performance of this risk assessment protocol, a second one was developed and now in phase of validation. The new risk assessment tool aims to be more specific, to help assessors (and farmers) to obtain a clear insight of weakness and strength of the farm.

Reviewer #3: The paper is a very well written and understandable document that presents interesting data related to Evaluation of a tool to identify risks for tail biting in pigs, that fits the Scope of PLOS One.

In my perspective the study has some main limitations that should be improved by the authors:

- Please provide the assessment protocol (Supplementary material) and, more important, provide the typology of the answers to each evaluation made (e.g. qualitative, score, presence/absence....etc.)

AU: The assessment protocol is provided in the supplementary material, as it is too lengthy to include as part of the main manuscript. We have provided additional information regarding the nature of the data collected (L118-120, L130, 132).

- Line 93 “designed to take a maximum of 15 minutes to complete per pen by a trained assessor (S1).”- Please add how many pens need to be evaluated per farm (it is written after, but I think that it should be also add here)(line 94-100)

AU: The sentence regarding the number of pens assessors needs to assess was moved as suggested, now lines 95-106

- Line 98 - How the assessor obtained the final weight achieved in the pen?

AU: The final weight reported on the risk assessment tool was obtained by consulting the producer. This information has been added at L110

- Line 100 101: How the assessor considered the vaccination protocol to be appropriate? Plesae clarify

AU: The assessments were carried out by private veterinary practitioners who were familiar with the farm (L95 – 96). Thus they were aware of the vaccination protocol in place.

- Line 106: Evaluation of physical and behavioral welfare indicators. It is not clear how this evaluation was performed for each variable assessed (e.g. score for each injuries or presence/absence?). Please clarify.

AU: The evaluation of physical and behavioural indicators is reported from L122-126. The level of severity of lesions was not considered, only presence or absence.

- Line 116: “Estimate the level of risk of tail biting in relation to six risk categories.” This output it is extremely subjective. There was any score used to support the assessors? Please clarify

AU: Yes, we agree that this is extremely subjective. We have clarified that whether the assessor considered there to be an overall risk, and what the level of risk was relative to each risk category, was based upon the outcome of the assessment, using their judgement (L130 – 132). We have included commentary in the discussion regarding how this is likely one of the reasons why the risk assessment tool is not effective (L549 – 552).

Line 120: “In each farm the assessor was asked to assess six pens; two at first stage weaner (from weaning day to approximately four weeks after weaning), two second stage weaner (from approximately four weeks after weaning to moving pigs to the finisher stage), and two finisher pens.” How the assessor selected the pens? The assessor evaluate the six pens in the same day? The risk estimated is a general risk per farm or per stage of production? The finisher pigs are the ones observed at abattoir? The pigs evaluated at the weaning stage are not the ones evaluated at abattoir? I’m asking this because the majority of tail biting outbreaks occurs at this stage and consequent severe tail loss with healing may be seen at abattoir. If these animals are not followed to the abattoir, some correlation analysis may failed. Please clarify these questions. Please explain the relationship between the batch of pigs sent to the abattoir with the assessed pigs at farm level

AU: The pens were selected randomly by the assessors and the farmer.

All pens were evaluated on the same day (L105-106).

As you point out, it was impossible to evaluate tail skin lesions at slaughter from pigs at the weaning stage, only at the finisher stage. Although it is true that tail-biting outbreaks occur more at the weaning stage, it is also reported that pigs at the finisher stage may also be prone to such abnormal behaviour (EFSA, 2007; EFSA AHAW, 2012), and it is recognised that the examination of tails at slaughter is a useful tool to identify welfare indicators on the farm. When performing the statistical analysis, this output was consider as well.

- Line 172 - Define representative sample

AU: The sentence was changed for clarification (L197-199).

- Line 185 - Please define: severe tail loss with healing

AU: Now defined in L214 – 216.

- Figure 1: Photos from score 0, 1, 2 and 3 are not original. Please add the source

AU: The source is in the text (line 215-217).

Line 486 - “ Our results indicate that the prevalence of pigs with the investigated injuries was low, and that visible injuries were not influenced by the production stage, or the number of enrichment materials provided per pen”. Please add some discussion regarding the fact that the majority were docked pigs which may have influenced the result you found. Please add the same information to the conclusions.

AU: The paragraph was deleted as in the updated results we did not report the prevalence of pigs with visible injuries. As such the paragraph was modified accordingly (L 581-594).

Line 554 “ Limitations associated with the implementation of the tool include the relative inexperience of the assessors, and the challenge presented by recording animal indicators from outside of pens”

AU: This has been added to the conclusions, L616-629.

Line 520 - “The lack of association between the two again suggests that in its current deployment, the application of the tool was not effective at identifying risk in general, even without considering the six specific risk categories”. Please add some information/discussion about the possible bias if pigs with severe tail lesions at farm could have been euthanised or not included in the batch assessed at abattoir level.

AU: This has been added to the discussion, L608-611.

Since Assessment of tail biting it is a topic of major importance in pork production chain, this submission may be suitable for publication, if authors improve the limitations aforementioned.

**Reviewer #4:** This study is very valuable because a significant reduction in the risk of tail biting can be achieved on many farms through the systematic evaluation and modification of management practices. The study aimed to validate a novel tail-biting risk assessment protocol by examining its association with tail lesion scores observed at slaughter. The authors indicated the inefficiency of the assessment tool in identifying actual risk, on farm. It underscored the need for enhanced training, and improvement at farm level in areas such as stocking density, enrichment provision and reducing tail docking, which are practical working solutions.
