# Supplementary material for: Evaluation of a scheme to identify risks for tail biting in pigs
Source: PLoS One. 2024 Aug 29;19(8):e0305960. doi: 10.1371/journal.pone.0305960 (PMC11361435; doi:10.1371/journal.pone.0305960)
Supplement: Attachment — Submitted filename: Response to reviewers.docx [file pone.0305960.s003.docx]

In the revised version, the authors provide more information and detail (esp. Supporting Information is valuable to understand the protocol), and the results are better documented. The conclusion has been much improved, with some suggestions how to improve the tool.

AU: We thank the reviewer for their comments, and appreciation of how we have improved the paper

However, some of the author’s responses to reviewers’ comments raised eyebrows; it really underscores that the assessors were not properly trained and the results issued from the application of the tool are not standardized at all.

«It transpired that the assessors used the levels in a different way than instructed» Similarly, “Assessors were consulted, and they informed the authors that they considered both level one and 2 to imply that there was minimal risk for tail biting in the pen. Levels 3 and 4 were being applied interchangeably whenever a risk factor related to that risk category could be clearly identified”. Risk categories: “However these were inserted by an external party who was also involved in the development of the tool, without piloting the definitions with either potential assessors or other personnel involved in the tool development”

So assessors just decided upon themselves to use the levels interchangeably: these issues should have been reported by the assessors and the procedure adapted accordingly during the study.

AU: We apologise for the lack of clarity as to why the study was carried out in the way it was. The aim was to evaluate the first Irish national tail biting risk assessment scheme. The risk assessment tool that was used as part of the scheme was developed by the Department of Agriculture, Food and the Marine, to meet the requirement by the EU that all member states should put in place a system of risk assessment for tail biting on farm. Following the tool development, and without any on farm testing, a scheme was established so that producers could have the assessment performed free of charge by their PVP (who were paid for their time by funding delivered via Animal Health Ireland (AHI)). The scheme was also managed by AHI, an industry-led, not-for-profit partnership between livestock producers, processors, animal health advisers and government. AHI co-ordinated and delivered training for the assessors, and stored the results of the assessment tool for each farm in a database. The role of the researchers in this project was simply to assess the effectiveness of the scheme in assessing risk for tail biting, and if it wasn’t effective, to try to understand why. The reviewer is completely correct, in that what we found is that the training was not sufficient to ensure that all the assessors were using the system in a consistent manner. This is partly because of the way the tool was designed, with as it turns out a lack of clarity as to how to assign risk level. However it was beyond the remit of the research team who ran the study to change procedures, or the definitions in the tool, our role was simply to gather and compile data, and analyse and report it. It was also not clear until we obtained the audit reports and examined the data where the problems lay. We have made some changes to the abstract, introduction, and discussion so that this aim is clearer.

It has to be made clear already in the abstract that the results have to be taken with a grain of salt, and that the tool lacks standardization and the assessors lack proper training. Readers should already know before they read the main text of the article that the authors tried to implement the tool but faced major issues and that they offer recommendations for those developing such tools in other countries. Knowing this beforehand avoids wasting readers’ time when they expect clear results.

AU: We have edited the text in the abstract in line with your suggestion.

“due to the poor performance of this risk assessment protocol, a second one was developed and now in phase of validation. The new risk assessment tool aims to be more specific, to help assessors (and farmers) to obtain a clear insight of weakness and strength of the farm.”

This raises the question if it would not have been better to wait for those results before publishing. There should be a clear benefit in publishing the failed tool for other researchers! Again, it should be highlighted in the abstract.

AU: The new tool is completely different, and has been developed from scratch, it is not an adaptation of the existing one, so the two are not comparable. We are planning to publish the results from our assessment of this new tool in a subsequent paper once the research is complete. We consider that the results from this study will be beneficial to other researchers, because it highlights ‘what not to do’, which is essential knowledge to help both researchers and policy makers in avoiding future mistakes. There have now been hundreds of assessments carried out using this tool by PVPs across Ireland, so a considerable amount of time and finances have been invested in the scheme, which is not providing the benefits that it aimed to.

Overall, the results are still a bit difficult to understand and it is not clear what authors exactly want to show.

AU: We understand that there is a lot of description, so we outlined the four aims of the analysis in the statistical analysis section (L277-281), and used the same headings in the results.

Table 7: I do not understand this table: if this is in a sample of pens where there was no risks then why is there a line with major risks.

AU: Assessors were asked to state whether they considered a pen was at risk or not overall (L135) as well as considering the level of risk that they considered each pen had in each of 6 risk categories (L136-142). It transpired that even when assessors considered that there was no overall risk, they sometimes still assigned levels of risk within the categories. Table 7 considers only the pens where the assessors considered there was no overall level of risk, and provides a breakdown of how they assigned the level of risk for each risk category. We have edited the text in this section (L385-390) to try and improve the explanation of what is in the table.

Figure 6: in the method there are now 3 levels: 0 being when the risk was not observed, then low risk and high risk. Here I suggest changing 0 and 1 to low and high if that is what it refers to.

AU: We apologise that the legend may have been misleading, we had updated it in the text, but not on the figure itself, which we have now done. For this, we only considered whether the assessors considered the pens at risk or not at risk overall (as per L135). We did not consider the level of risk assigned to each of the risk categories.

L 290: suggestion: random effect instead of repeated effect.

AU: We considered it a repeated effect because we were repeating the measurements within the experimental unit (the farm). However, repeated effects in linear mixed models are treated as random effects so they are computationally the same
