# Supplementary material for: Evaluation of a scheme to identify risks for tail biting in pigs
Source: PLoS One. 2024 Aug 29;19(8):e0305960. doi: 10.1371/journal.pone.0305960 (PMC11361435; doi:10.1371/journal.pone.0305960)
Supplement: Attachment — Submitted filename: Response to reviewers.docx [file pone.0305960.s004.docx]

Lines 256-260: methodological question. The risk on doing this pen level estimate is that some pigs had a high number of lesions. Do you have a measure of disperson (Standard error), or do you work with the median or some parameters which avoids not considering the variation in data? I am not sure if this is reflected in your results in Table 8, since the interquartile range you provide is already of pens not of individuals within a pen.
Another variable, ‘Visible injuries’ was created by summing the number of tail, ear, flank and aggression lesions in the pen, and dividing by the number of pigs per pen to provide a pen level estimate relative to the number of pigs

AU: We agree with the problem you have identified, thank you for this important observation. It is possible that either a small number of pigs had a lot of lesions, or many pigs had a low number, and there is no distinction between which is the case. However, unfortunately the data that the assessors collected did not go into this level of detail, all that was on the report was the total number of lesions that they saw in the pen, so it was not possible to calculate the median lesion number per pig. Because all we could do was calculate the mean (no. lesions/no. pigs / pen) this is the value we used in the analysis, which was carried out using a non-parametric test. We added some text from L572-576 to acknowledge this issue.

Line 291. It seems that pens were only observed once, why is it used as repeated effect?
AU: Yes, each pen was only observed once, but we considered the farm the experimental unit, so each pen was a repeated measure within each farm

Line 355. For the undocked pigs, it is understood that you used the same scoring system at the abattoir? Do you think you should use in futur studies a different one, which could evaluate lenght?
AU: This refers to L255 I think. There was only one scoring system because only a tiny proportion of pigs in Ireland are not docked. In the study population it was less than 1%. However yes, we agree that in future on populations of pigs where the tails are not docked a measure of length would be very useful!

Lines 571-578. Take into account my previous comment on the fact that when you score at pen level injuries, one biased factor could be disperion (ie animals heavily injured increasing the overall score)
AU: See answer above

Line 614- 625. In my opinion, this problem about lack of correlation between on farm risk assessment and at abattoir final result could be worse in undocked pigs. Your sample size is probably not sufficient to separate both types of pigs, but it would be an interesting study for the study. Not only because tail biting is still more problemàtic in undocked pigs, but because the scoring system at the abattoir needs to be able to disentangle those pigs which has suffered tail biting but lesion has healed over time.

AU: We agree, this would be in interesting research question for a future project. However at the moment there are not sufficient pigs in Ireland where tails are not docked to carry out this evaluation. We have added some text to L640-643
